# Policy protection for mental illness in China from the perspective of policy tools

**DOI:** 10.3389/fpubh.2025.1503742

**Published:** 2025-05-08

**Authors:** Qingli Tan, Hongrui Cui, Wanyi Huang, Jiaying Peng, Daiheng Lin

**Affiliations:** ^1^School of Medical Business, Guangdong Pharmaceutical University, Zhongshan, China; ^2^Guangdong Research Base for Drug Regulatory Science, Guangzhou, China; ^3^NMPA Key Laboratory for Technology Research and Evaluation of Pharmacovigilance, Guangzhou, China; ^4^School of Nursing, Guangdong Pharmaceutical University, Yunfu, China

**Keywords:** mental health policy, mental disability protection, policy tool analysis, three-dimensional policy framework, mental health service providers

## Abstract

**Objective:**

To analyze the policies related to mental illness in China, concentrate on the deficiencies in the configuration of policy tools, and to provide reference suggestions for the government to improve the mental illness protection policies.

**Methods:**

Using the policy tools method, we constructed a three-dimensional model of “policy tools – service providers – policy objectives,” and statistically analyzed the characteristics and reasonableness of the configuration of policy tools in each dimension and the cross-fertilization of dimensions.

**Results:**

The dimension of policy tools has the largest proportion of environmental tools, 46.71%. With 28 and 25.29% of supply side and demand side tools. The public health sector has the largest proportion of service-providing entities, 46.82%; and the policy objectives dimension has the largest proportion of social support, 71.35%. The policy tools used by each service subject are biased toward the environmental tools, with less application of demand side and supply side tools, and insufficient coordination of policy tools. The distribution of policy tools and objectives in existing policies is uneven, with many policy programs but little guidance, and uneven allocation of resources in policy implementation, leading to poor results.

**Conclusion:**

China should pay more attention to social support and protection, and suggests increasing the proportion of supply side and demand side tools. Public health sectors should strengthen the training of medical personnel with mental disabilities. Government will increase the investment in the construction of community medical institutions and optimize the financing model of medical insurance and the distribution of medical resources to strengthen the function of family security, and safeguard the needs of patients with mental disabilities.

## 1 Introduction

With the development of China’s socio-economic and pharmaceutical industries, more and more difficult and complicated diseases have been effectively controlled, but mental illnesses are on the rise, and the lifetime prevalence of mental illness in China is as high as 16.6% (1). As of 2017, there were more than 240 million people suffering from mental disorders in China, with more than 16 million people suffering from 6 types of severe mental disorders, led by schizophrenia (2), mental health problems have become an important issue in the field of health, which protection policy for the development of China’s society and people’s livelihood has become increasingly important, the scientific policy can be more effective in guiding and standardizing the prevention, treatment and protection of mental disorders. Taking the 2013 Mental Health Law as the demarcation point, China’s local legislation on mental health has gone through a “pre-mental health law” and a “post-mental health law” stages (3). Since the management of severe mental illness has been included in basic public health services, China’s mental health work has entered a period of rapid development (4). It is necessary to increase policy support, provide more fair, accessible, systematic and continuous basic rehabilitation services for patients with mental disorders, and carry out a three-year national community rehabilitation service integration action for mental disorders (5). Although today’s domestic mental health policies are comprehensive in their coverage, there are a number of pressing issues that need to be addressed. There are very few studies that analyze documents on mental health based on the perspective of policy tools. This paper takes the protection policy of mental illness as the theme, utilizes the policy tool method, constructs the three-dimensional model of “policy instruments-service providers-policy targets,” quantifies the data and constructs charts from different aspects, describes and presents the policy content more intuitively and deeply, which is important and practical significance for the interpretation and implementation of the policy. It is of marked practical significance to the interpretation and implementation of the policy. This paper selects a total of 45 policy issued by China from 2008 to 2022, and analyzes them from the three dimensions of policy instruments, service providers, and policy objectives, to explore the key points of the existing mental illness protection policy and analyze and summarize its problems, so as to provide reference suggestions for the government to solve the existing deficiencies and to formulate and improve the mental illness protection policy.

## 2 Literature review

There have been few studies related to mental health policy in China and they have mainly concentrate on the overall legislation of the policy, its utilization, and have been limited to community services, countermeasure studies in a certain region, etc., and there have been relatively few studies on the analysis of the policy of the overall system.

Li et al. (6) combined the relevant literature on mental health policy research with interviews to categorize China’s mental health-related policies from 1949 to 2009 into three phases; 1949–1961 was the phase of China’s rapid mental health development, 1962–1978 was the phase of mental health development from wandering to steady development, and 1978–2009 was the phase of rapid mental health service development and reform stage, and it argued that at that time, China’s mental health service resources were in short supply, the psychiatric treatment rate was low, and there were still many aspects that needed to be improved. Chen et al. (7) analyzed and summarized China’s important mental health policies from 2009 to 2019, and concluded that the prevention and treatment of mental illness should be strengthened while improving the policies again. Ma et al. (8) use the method of participant observation believes that the 686 program is an important direction for the reform of China’s mental health service model, a government-led, community-based continuous prevention and treatment model, laying a solid foundation for the combination of hospital community assistance. Through a combination of literature and field research, Li (9) examined the social security needs and supply of persons with mental disabilities, and concluded that although the basic rights of persons with mental disabilities, such as the right to life, are better protected, the existing policy pays insufficient attention to their right to development and participation. Gao et al. (10) use literature analysis method and believe that China’s government intervention as the main form of response to serious mental disorders, to carry out a number of relief and rescue work, but the form of relief and rescue: relief and rescue in a single way, it is difficult to meet the daily needs of patients.

There is a serious shortage of mental health resources in China, and they are unevenly distributed. Studies by Li shown that in 2009 there were significant regional differences in the number of psychiatrists in China, and in some areas there were even gaps in services and a lack of professional mental health service providers (6). Ma et al. (11) also pointed out that there is still a large gap between the number of psychiatric hospitals in China. The number of psychiatric beds and the average level of mental health resource allocation in high-income countries still have a large gap. Xin et al. (12) believe that future research should focus on the impact of factors such as age, economic status, participation in free community medication services, and diagnosed disease types on medical expenses.

China’s mental health policy tools have structural imbalances. Chen et al. (13) showed that the internal structure of supply-type policy tools is unreasonable and difficult to meet the development needs of mental health services. Gao et al. (14) also mentioned that the use of environment-type, supply-type and demand-type policy tools in mental health policies is unbalanced, which further confirms the problem of structural imbalance in policy tools. Liu (15) suggested balancing the structure of policy tools, increasing investment in resources such as talent, finance and infrastructure, and avoiding internal imbalances in supply-type policy tools.

In summary, research on mental health policies mainly uses a literature research method and mainly focused on policy contents and usage. Most studies of policy tools use two-dimensional models. The research has been limited to community services and studies of policy responses in a specific region. There have been relatively few studies on the overall analysis of policies, lack of comprehensive analysis of the protection and assistance policies for people with mental illness. Therefore, this study uses the policy tools and construct the “policy tool-service providing-policy goal” three-dimensional model, show the overall situation of China’s policy protection for mental illness in a more detailed and intuitive way, and analyze the deficiencies of the existing policies, so as to provide reference suggestions for the government to formulate and improve the policy system of protection for mental illness, and to help people with mental illness to have a more comprehensive policy of protection.

## 3 Source and methods

### 3.1 Source of policies

In the official website of the Chinese government, the official website of the Ministry of Civil Affairs, the official website of the National Health and Health Commission, the official website of the China Disabled Persons’ Federation, and the official website of the China Center for Disease Control and Prevention, we searched for policy documents containing the above keywords, such as “mental health,” “mental disability,” “mental disorder,” and “mental health,” “mental health,” “mental disability,” “mental disability,” “mental disorder.” The search was conducted on March 28, 2008, and the results were retrieved on December 29, 2022. A total of 2,925 documents containing the above keywords were obtained through full-text search. Most of documents are invalid, such as work guidelines and political reports. In order to improve the pertinence and accuracy of the research policy in this paper, the retrieved documents were manually read and screened.

The inclusion and exclusion criteria are: Inclusion of formal policy documents related to the protection of mental disabilities issued by the State Council, ministries and commissions, and national organizations such as the Federation of Disabled Persons. Exclusion of informal documents, such as letters in response to proposals. Exclusion of the sentence “to strengthen,” “to reinforce” with no specific methods.

After checking the documents twice, we confirm 45 policy documents were finally included. Part of documents are shown in Table 1.

**Table 1:** Summary of policy documents.

Serial number	Issuing authority	Name of the document
1	Former Ministry of Health, Ministry of Education, Ministry of Public Security, Ministry of Civil Affairs, Ministry of Justice, Ministry of Finance, China Disabled Persons’ Federation	Circular of the General Office of the State Council Transmitting the Guiding Opinions of the Ministry of Health and Other Departments on Further Strengthening the Work of Mental Health (General Office of the State Council [2004] No. 71)
2	Former Office of the Ministry of Health	Circular of the General Office of the Ministry of Health on the Issuance of the Implementation Requirements for the 2007 Centrally Subsidized Local Management and Treatment Program for Serious Mental Diseases and the Rural Epilepsy Prevention and Management Program (Office of Health and Disease Control [2008] No. 54)
…	…	…
42	Ministry of Civil Affairs, General Administration of Market Regulation	Opinions of the Ministry of Civil Affairs and the General Administration of Market Supervision on Comprehensively Promoting Civil Affairs Standardization in the New Era (Ministry of Civil Affairs [2022] No. 58)
43	General Office of the National Health Commission	Circular of the General Office of the National Health Commission on the Issuance of Service Specifications for Autism Screening and Intervention for Children Aged 0 to 6 (for Trial Implementation) (National Health Office for Women and Children [2022] No. 12)
44	Ministry of Civil Affairs Ministry of Finance China Disabled Persons’ Federation	Opinions of the Ministry of Civil Affairs and the Ministry of Finance of the China Disabled Persons’ Federation on Strengthening the Precise Management of the Two Subsidies for Persons with Disabilities (Ministry of Civil Affairs [2022] No. 79)
45	Ministry of Civil Affairs Ministry of Finance National Health and Health Commission China Disabled Persons’ Federation	Notice of the Ministry of Civil Affairs and the Ministry of Finance of the National Health and Health Commission of the China Disabled Persons’ Federation on the implementation of the “Jingkang Integration Action” (Ministry of Civil Affairs [2022] No. 104)

### 3.2 Methods

#### 3.2.1 Three-dimensional framework

**Figure 1:**
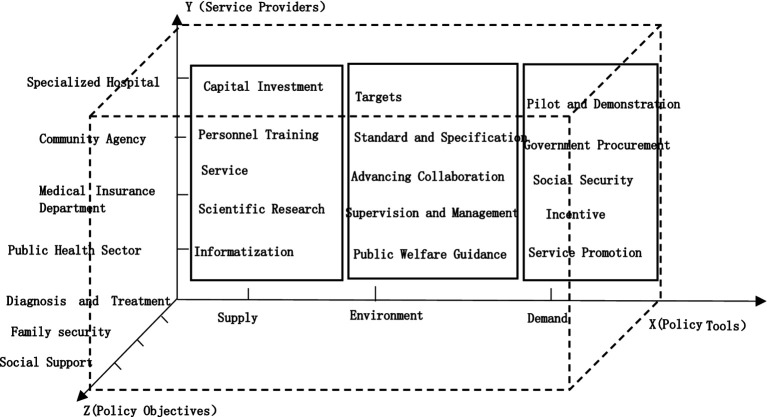
Three-dimensional model stereogram.

A rational structure of policy tools is a prerequisite for the realization of policy effects. In order to better explore the intrinsic connection of the mental illness protection policy, this paper introduces another two dimensions of policy analysis on the basis of Rothwell and Zegveld’s policy tools: the dimension of the service providers and the dimension of the policy objectives. The study of service providers is favorable to explore the effectiveness of policy implementation. What factors influence policy implementation. Studying the dimensions of policy objectives is conducive to clarifying the focus of policies. It further shows the configuration of policy tools for China’s mental disability protection, and provides suggestions for the government to formulate and improve the mental illness protection policies. The three-dimensional model is shown in Figure 1.

##### 3.2.1.1 Dimension X: policy tools

This study adopts Rothwell and Zegveld’s classification of policy tools. The policy tools are categorized into three types: supply, environment and demand (16). The methodology has been widely used in the field of policy analysis and provides a better representation of the role of different policy tools (17). Supply-type policy tools refer to the direct supply of resources by the government through financial input, personnel training and other means to provide motivational support for the protection of mental disorders (18). Environment-type policy tools refer to the indirect means by the government through standards and specifications, supervision and management or other means to create a favorable environment for the protection of mental disorders (19). Environment-type policy tools is mainly played a role in influencing. Demand-type policy tools refer to the means by which the government through government purchasing, service promotion or other means to stimulate demand and increase the motivation of service providers (20).

##### 3.2.1.2 Dimension Y: service providers

The development of mental illness protection is inextricably linked to the service providers who undertake this task. Through the 45 policies analysis, the main service providers related to China’s mental illness protection policy can be divided into the public health sector, medical insurance department, community agency and specialized hospital.

##### 3.2.1.3 Dimension Z: policy objectives

Policy objectives are the effects and values that policymakers want to achieve in the process of governance (21). Through the 45 policies analysis, policy objectives can be divided into diagnosis and treatment, family security and social support.

#### 3.2.2 Coding of policy tools

For statistical and research purposes, we use Microsoft Excel (2019 version) code and summarize the 45 policy documents. First, policies are coded in ascending order according to the date of promulgation. Second, the chapters and appendices in each policy are coded, followed by the sections and subsections under each chapter, and finally the sentences in the policy that meet the screening criteria are coded. If there is no subsection, the last digit indicates the sentence code. For example, 45–2–5-2(1) in Table 2 refers to the first sentence 45–2–5-2(1) of the second subsection in the fifth section in the second chapter of policy NO.45: “Guide the collection and use of social funds.” 2(1)-5–1-1 refers to the first sentence of section 1 of chapter 5 of Appendix 1 of policy NO. 2: “Each province shall formulate a detailed project implementation plan (including a plan for the use of funds), a supervision plan, and take the necessary measures to ensure that funds are fully and timely allocated to the project implementation unit.” If the same policy document contains multiple policy tools, they are counted separately. The coding of policy tools is shown in Table 2.

**Table 2:** The current situation of policy tool allocation at all levels of mental illness protection policy in China.

Type of policy tools		Name of policy tool and number	Frequency/composition %	Proportion%	Sum %
Supply	Capital Investment	1–3–1-2, 2 (1)-5–1-1, 2 (3)-5–1-1, 3–3–10-1, …….45–3–1-4	64/15.50	4.34	28.00
	Personnel training	1–4–1-2, 1–5–2-4, 2 (1)-2–1-2, 2 (1)-4–1-2, …….45–2–5-2 (1)	76/18.40	5.15	
	Services	1–3–2-6, 1–4-5, 1–5-1, 1–5–2-2, …….45–3–1-5	155/37.53	10.51	
	Scientific Research	1–4–2-3, 1–7-1, 6–3–3-6, 6–12–2-3, …….39–3–2-3	36/8.72	2.44	
	Informatization	1–7-2, 2 (1)-3–3-1, 2 (3)-4–2-1, 3–3–13–2, …….45–3–1-6	82/19.85	5.56	
Environment	Targets	1–1–1-1, 1–1–1-2, 1–2–1-1, 1–3–1-1, …….45–2–4-3 (1)	86/12.48	5.83	46.71
	Standards and specifications	1–3–1-3, 1–3–2-3, 1–3–2-5, 1–5–2-3, …….45–2–6-5 (2)	320/46.44	21.69	
	Advancing Collaboration	1–3–2-2, 2 (3)-2–1-1, 2 (3)-2–2-1, 3–1–2-3, …….45–3–1-3	80/11.61	5.42	
	Supervision and management	1–5–3-3, 1–6-2, 1–8-3, 1–8-4, …….45–3–1-4	155/22.50	10.51	
	Public Welfare Guidance	1–3–1-3, 3–2–5-4, 3–3–13–3, 6–10–1-4, …….45–2–5-2 (2)	48/6.97	3.25	
Demand	Pilots and Demonstrations	2 (1)-2–1-1, 2 (1)-2–1-2, 2 (1)-3–2-1, 3–2–6-4, …….45–3–4-2	33/8.85	2.24	25.29
	Government Purchasing	2 (1)-5–3-4, 4–3–2-9, 6–6–1-4, 9–4–7-8, …….45–2–5-2 (1)	37/9.92	2.51	
	Social security	1–3–2-4, 1–4–2-2, 1–4-4, 1–5–3-6, …….45–3–1-8	102/27.35	6.92	
	Incentives	3–2–5-4, 3–2–6-7, 3–3–10-3, 3–3–13–3, …….45–3–2-2	91/24.40	6.17	
	Service Promotion	1–3–3-2, 1–4–1-3, 1–4-3, 1–6–2-2, …….45–2–6-2 (3)	110/29.49	7.46	

## 4 Results

### 4.1 Single dimensional analysis

In policy tools dimension (X), environment type policy tools (*n* = 413, 46.71%) accounted for the highest proportion, followed by supply type policy tools (*n* = 373, 25.29%), which shows that the government is more concerned about policy guidance and capital investment. Among environment type policy tools, the government uses the most policy tools of standards and specification (*n* = 320, 21.69%), and the least public welfare guidance (*n* = 48, 3.25%). This result is in line with the Chinese government’s requirements for the standardization of industry management. Among supply type policies, the government most often uses service (*n* = 155, 10.51%), and the lowest proportion is scientific research (*n* = 36, 2.44%). However, this does not mean that the government does not value scientific research on mental illness. Rather, the government can provide direct support for services related to the recovery from mental illness. Among demand policy tools, service promotion (*n* = 110, 7.46%) account for the largest share. It means that the government hopes to play a greater role in the rehabilitation of people with mental illness, relieve their financial pressure, and provide more social security for them.

In terms of the main service providers (Y), the public health sector, community agency, specialized hospital and medical insurance department accounted for 46.82, 36.91, 13.80 and 2.47%, respectively. This shows that public health departments and community institutions are the main service providers of China’s mental illness protection policies, such as 22–1–1-1 and 22–1–5-2. It can be seen that the current medical insurance policies are very inadequate in supporting mental illness, and also reflects that the functions and roles of the medical insurance department have not been fully utilized. For example, the medical insurance department should investigate the medical treatment rules for mental illness. So that solve the problem that the current medical insurance payment methods are not diverse enough (22).

In the policy objective dimension (Z), social support, diagnosis and treatment, and family security accounted for 71.35, 26.42, and 2.22%. The objectives of mental illness protection policies mainly rely on social support. Also it can be seen that the government does not attach enough importance to the function of family security in the recovery process of mental illness. In policy documents (e.g., 19-2-3), it is usually in the form of an appeal to ask families to pay attention to preventing mental illness and caring for people with mental illness. But have no specific measures are proposed. It can also be understand that the government should not intervene in family affairs excessively.

### 4.2 Bi-dimension cross analysis

#### 4.2.1 X–Y dimension

As shown in Table 3, the proportions of service providers corresponding to the three policy tools, from high to low are all the same: public health sector, community agency, specialized hospital, and “medical insurance department.” This result is the same as the result of the Y-dimension analysis, which reflects the dominant position of the national public health sector (*n* = 1,157, 46.21%) and community agency (*n* = 942, 37.62%) play an important role in providing support service (*n* = 113, 4.51%) for mental illness rehabilitation and establishing standard and specification (*n* = 263, 10.50%).

**Table 3:** Statistical results on the configuration of three-dimensional policy tool.

X-dimension (Secondary policy tools)	Y-dimension				(Grand) total	Z-dimension			(Grand) total
	Specialized hospital	Community agency	Medical insurance department	Public health sector		Social security (pensions, medical insurance)	Family security	Diagnosis and treatment	
Capital investment	4	24	4	58	90/3.64%	58	2	15	75/4.07%
Personnel training	53	27	0	34	114/4.62%	38	2	62	102/5.54%
Services	64	113	5	106	288/11.58%	132	5	92	229/12.43%
Scientific research	23	8	0	20	51/2.07%	19	0	26	45/2.44%
Informatization	13	49	1	76	139/5.63%	78	0	17	95/5.15%
Supply type total	157/23.02%	221/32.40%	10/1.47%	294/43.11%	682/27.24%	325/59.52%	9/1.65%	212/38.83%	546/29.63%
Targets	21	35	5	77	138/5.59%	83	3	41	127/6.89%
Standards and Specifications	61	263	19	238	581/23.62%	294	7	72	373/20.23%
Advancing collaboration	17	33	3	66	119/4.81%	65	5	29	99/5.37%
Supervision and management	17	102	4	143	266/10.76%	144	1	20	165/8.96%
Public welfare guidance	2	26	0	39	67/2.69%	48	1	7	56/3.04%
Environment type total	118/10.05%	459/39.10%	34/2.89%	563/47.96%	1174/46.88%	634/77.32%	17/2.07%	169/20.61%	820/44.49%
Pilots and demonstrations	2	16	1	32	51/2.07%	26	0	9	35/1.89%
Government purchasing	3	14	2	34	53/2.12%	36	1	10	47/2.56%
Social security	19	72	14	70	175/7.08%	99	8	33	140/7.60%
Incentives	15	49	3	71	138/5.59%	88	2	24	114/6.19%
Service promotion	27	81	0	93	201/8.13%	107	4	30	141/7.65%
Demand type total	66/10.19%	262/40.43%	20/3.09	300/46.29%	648/25.88%	356/74.63%	15/3.15%	106/22.22%	477/25.88%

Combined with the corresponding target (*n* = 138, 5.59%) and supervision and management (*n* = 266, 10.76%) of Y-dimension, the Chinese government is more prefer to formulating policies to improve the current situation of mental illness and protect the interests of patients. The results are specialized hospital-capital investment (*n* = 4, 0.16%), specialized hospital-government procurement (*n* = 3, 0.12%) and medical insurance department -personnel training (*n* = 0, 0%) and medical insurance department -scientific research (*n* = 0, 0%), which mean there are many policy objectives and plans but few specific measures. However, this makes it difficult for policies to be effective.

#### 4.2.2 The X–Z dimension

The policy objectives corresponding to the different policy tools. As shown in Table 3, the policy objectives corresponding to the three policy tools in descending order are: social support, diagnosis and treatment, and family security. This result is the same as the result of the Z-dimension analysis. Environment-type policy tools (*n* = 820, 44.49%) still account for the highest proportion. Among the supply-type policy tools, personnel training -diagnosis and treatment (*n* = 62, 3.36%) is greater than personnel training -social support (*n* = 38, 2.06%), and scientific research-diagnosis and treatment (*n* = 26, 1.41%) is greater than that of scientific research—social support (*n* = 19, 1.03%), The results indicate that the government is more inclined to directly provide assistance in talent and scientific research which require long-term investment and high costs. Rather than relying primarily on market and social supply. Among social support, standard and specification-social support” (*n* = 294, 15.95%) has the highest number. Due to the limited financial resources and low degree of specialization of civil society and non-government organizations, and the enterprises pursue profits. So there are norms must be adopted to heal and care for the rehabilitation of people with mental illness.

#### 4.2.3 Y–Z dimension

**Figure 2:**
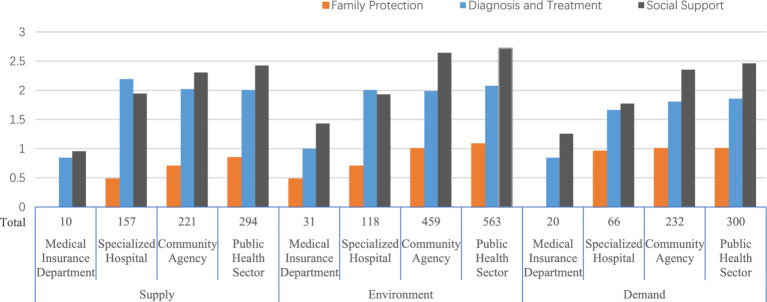
Three-dimensional policy tools configuration histogram.

Figure 2 and Table 4 show that there are significant differences in the proportion of policy objectives corresponding to different service providers. Public health sector-social support (*n* = 1,084, 33.97%) and community institutions-social support (*n* = 867, 27.17%) far exceed those of the other values in the Y–Z dimension. This shows that the government pays more attention to rehabilitation than to the treatment of mental illness. Among the demand-type policy tools, the number of specialized hospitals – diagnosis and treatment (*n* = 46, 1.44%) is smaller than that of specialized hospitals – social support (*n* = 59, 1.85%). This anomaly shows that the government attaches importance to social forces in improving the rehabilitation of mental illness. It can be summarized as follows: the government regards specialized hospitals as the main institutions for treating mental illness. However, compared with treatment, the government pays more attention to guiding the public health sector through policies to introduce social forces to assist primary medical institutions in the rehabilitation of mental illness patients.

**Table 4:** Statistical results of three-dimensional policy tool configuration (Y-Z).

	Social support	Family protection	Diagnosis and treatment	(Grand) total
Specialized hospital	232	17	302	551/17.27%
Community agency	867	25	265	1157/36.26%
Medical insurance department	52	2	24	78/2.44%
Public health sector	1,084	29	292	1405/44.03%
Total	2235/70.04%	73/2.29%	883/27.67%	3,191

## 5 Discussion

### 5.1 X-dimension policy tools are highly skewed, with uneven policy distribution and structural imbalances

The above reasons may be attributed to the following aspects. First, there is a lack of supporting facilities for mental health services in China. Second, the poor effectiveness of policies formulated in the early stages has led to subsequent policies constantly patching up previous policies, resulting in an excessive number of policies with the same content. For example, Policy No. 11 mentions “vigorously leveraging the important role of community health service institutions and rural medical and health institutions in mental health work.” Subsequent policies also contain similar statements. But the reality is that due to a lack of policy management knowledge and clinical mental health expertise, the role of community and rural medical and health institutions is limited (23). Third, there is insufficient training of mental health professionals in China, inadequate policies, and few scientific research policies for mental disorders. In addition, the development of psychiatry departments in general hospitals is relatively weak, resulting in a lack of timely diagnosis and treatment of mental disorders. Fourth, demand-type policies account for the largest proportion of service promotion (*n* = 110, 29.49%). But the reality is that the publicity and promotion of policies is insufficient, resulting in patients and their families not understanding the relevant policy procedures. This makes some patients have low participation in mental health policies. Excessive creation of environment-type policies and lack of demand-type policies will lead to the development of mental health with strong policy support but overall lack of momentum.

### 5.2 Insufficient health insurance support

At present, the level of development of medical insurance in various provinces and cities in China is uneven, and there is a large urban–rural gap within each region. There are certain differences in the reimbursement ratios and maximum for medical insurance available to people with mental illness in different cities. Two of the most typical problems are the reimbursement ratios and maximum for urban workers’ medical insurance are better than those for urban–rural medical insurance, and the subsidies for inpatient treatment are higher than those for outpatient treatment. Due to their illness, most people with mental disabilities have difficulty finding employment and have a low income, which results in the reimbursement ratios and maximum for medical insurance for people with mental disabilities being generally lower. For the reason of particularity of the disease, most mental illnesses have characteristics such as a long course and complex causes. The costs incurred by long-term diagnosis and treatment are high.

In China’s medical security system, medical insurance is the mainstay and medical assistance is supplementary, they work together to provide medical security. The two are complementary. However, due to the difference in the overall planning level between the medical assistance system and the medical insurance system, the synergy of the two systems is weakened (25). China’s medical assistance system has problems with insufficient capital investment (*n* = 4, 0.16%) and inadequate service (*n* = 5, 0.20%), which limits its development and makes it less resilient to risks (26). Coupled with the uneven assistance policies in different regions and the large regional differences in subsidy, which is not conducive to alleviating the medical burden on people with mental disabilities. Difficulties faced by people with disabilities The overall standard of the nursing allowance in the disability living allowance and nursing allowance system for people with severe disabilities is low (27), and it is still insufficient to cover the nursing costs required by people with severe mental disabilities.

### 5.3 The government should pay attention to the role of the family security in the mental illness recovery

Some scholars divide family protection into “traditional family security” and “family social security” (28). The former mainly refers to the reciprocal mechanism among family members, in which the family provides family members with economic security, service security and spiritual comfort. The latter refers to a family protection policy implemented by the government for families, which is both economic and welfare-oriented. The relationship between the two is not antagonistic. On the contrary, “family social security” can promote the functioning of the security function within the family. Table 3 shows that the total number of family security is 41, accounting for only 2.22% of the total number of policy objectives. Both “traditional family security” and “family social security” are in a state of near absence. Social support, however, is as high as 71.35%.

On the one hand, the lack of “traditional family security” is related to the changes in family structure caused by the impact of modern industrial civilization (29) and the weakening of the family’s security function. On the other hand, the lack of “family social security” is mainly related to the lack of top-level design and relevant policies in the existing system, such as clear family support policies and laws, and the fact that a family-oriented welfare system has not yet been established. There is a substitution relationship between state security and family security. Increasing social security will replace or weaken some of the functions of family security, reducing personal dependence on the family. The original family security system gradually disintegrates, which also leads to the weakening of family functions. However, family security is not completely replaceable. Especially for people with mental disabilities, the role of the family is particularly important. The family is the most direct living carrier for people with disabilities. Family members can accurately grasp the type and degree of disability, special skills and potential, psychological status of the disabled, and provide them financial security, service security, and spiritual comfort in a targeted manner (30). However, in terms of policy structure, China currently places more emphasis on social support and security, and pays less attention to family security. China’s social security level is not high, and the overall situation lags behind the demographic structure (29). Without paying attention to the role of family security, it is difficult to improve the protection for people with mental illness. This can be seen in three areas: financial, domestic services, and emotional support. Financially, this is mainly reflected in the difficulties that disabled people face in finding employment and the heavy burden on their families. In terms of domestic services, the high care needs of people with severe disabilities cannot be met by the current system, which can easily lead to families reducing their output, thus further exacerbating their financial difficulties. Emotionally, due to various factors in reality, such as financial burdens and changes in family values, it is often impossible to provide adequate emotional support to patients.

## 6 Recommendations

### 6.1 Increase the use of supply-type policy tools

There is a shortage of talents in mental health in China, and it is difficult to provide adequate service support (31). The reason is the lack of government investment. Although the Mental Health Law of the People’s Republic of China stipulates that the treatment level of mental health personnel should be improved and appropriate allowances should be given, but the actual implementation has not been carried out (24). China’s government’s investment in mental health institutions has increased year by year from 2002 to 2019, and the overall trend of mental health resource allocation has been on the rise nationwide (32). However, the current mental health allocation is still unable to keep up with the growing demand for mental health, and in order to meet the demand, it is necessary to expand the mental health talent pool. Since 2011, the annual growth rate of the government’s financial investment has been slower than the average annual growth rate of the medical staff (32), which has led to a failure to raise the level of treatment for mental health personnel, and the allowances that they should receive have not been realized (24). In addition to low welfare benefits, the mental health profession also suffers from high occupational risks, poor working conditions, and a strong sense of social discrimination, which leads to a higher incidence of burnout among psychiatric medical personnel compared to other professions (33), and a higher willingness to leave the profession (34). The government should improve the treatment level of mental health personnel, such as by raising salaries, giving appropriate allowances or benefits to alleviate the shortage of personnel. At the same time, personnel training should be strengthened, and the government should provide certain technical support and collaborate with universities and hospitals to cultivate specialists.

Strengthen the construction of community rehabilitation and enhance the relevance of service organizations. Patients with mental disabilities, except for the acute stage requiring hospitalization, live in the community for most of the time, taking medication to control their condition, as well as for subsequent rehabilitation (25), and there is a greater demand in the community. However, the number of specialized institutions in the community is currently limited, which makes it more difficult for patients with mental disabilities to find receiving institutions, or to be mixed with patients with other types of disabilities in care without access to specialized services. In addition to the small number of institutions, the shortage of professional staff is also an important factor. Therefore, the Government should increase its investment in the construction of community and public organizations, such as technical support and financial investment. It can also encourage private institutions to specialize by giving certain preferential policies, such as lower taxes and policy subsidies.

### 6.2 Optimizing the use of environment-type policy tools

The proportion of standard and specification should be appropriately reduced, the proportion of other types of policy tools should be increased. The introduction of new media such as the Internet, social media, and mobile applications has become an important channel for the dissemination of public service advertisements. Compared with traditional media, new media platforms are more down-to-earth and are also more popular with younger Internet users, and the dissemination is relatively strong (35). The government should attach importance to and give play to the guiding role of public welfare, create a favorable social environment, and advocate for social forces to provide assistance to people with mental disabilities. Government departments should work together with social groups such as the China Disabled Persons’ Federation to reduce public prejudice and stereotypes against people with mental disabilities through public service advertisements, which will also facilitate the implementation of subsequent systems (36). Initiate public welfare activities and guide social assistance organizations or voluntary organizations to carry out activities such as sending warmth and public welfare fundraising to provide material or spiritual support to people with mental disabilities.

### 6.3 Increase the use of demand-type policy tools

Relevant government departments, such as State Administration for Industry and Commerce of the People’s Republic of China, National Press and Public Administration. Other social organizations, such as China Disabled Persons’ Federation and China Foundation for Disabled Persons, have jointly planned and financed public service announcements on television, and public service announcements can be made in the form of short videos, microfilms and other forms of public service promotion on the Internet. Local governments take the lead, and local public welfare organizations and schools respond by organizing activities such as lectures and contests with prizes, to enhance the general public’s understanding of persons with mental disabilities and create a good social environment for persons with mental disabilities.

### 6.4 Optimize the medical insurance system

The Government should speed up the process of integrating urban and rural areas in medical insurance and promote a balanced distribution of medical resources. It should improve the medical insurance system and set up special medical insurance for mental illnesses. Optimize the price of psychiatric medical services, and raise the reimbursement ratio and maximum of medical insurance for patients with mental disabilities. Improve the medical assistance system and enhance its risk-resistant capacity. Raise the level of coordination of the assistance system, strengthen the connection with the medical insurance system, play a complementary role, and safeguard the medical needs of patients (37). Reducing the medical burden on patients. Medication is the main and basic measure for improving mental disorders. Serious mental disorders, in particular, especially need long-term, reasonable medication to maintain treatment, and some even have to take medication for life, which brings a heavy economic and mental burden to the family and society (38). The government should further expand the number of psychotropic drugs included in the basic medical insurance catalog, increase reimbursement, and implement a medication subsidy system to protect patients’ medication needs and reduce their financial burden (39).

## 7 Limitations and prospects

The study is not free from limitations. Due to only policies at the national-level governments were searched. The results can only represent the allocation of policy tools at the national level. In order to further study the protection of policies for people with mental illness at the local governments, future research should select representative provinces and cities. Searching and analyzing their policies to obtain more valuable experience for promotion.

## Data Availability

The raw data supporting the conclusions of this article will be made available by the authors, without undue reservation.
